# Cysteine S-acetylation is a widespread post-translational modification on metabolic proteins

**DOI:** 10.1038/s44324-025-00081-2

**Published:** 2025-11-07

**Authors:** E. Keith Keenan, Akshay Bareja, Yannie Lam, Paul A. Grimsrud, Matthew D. Hirschey

**Affiliations:** 1 Duke Molecular Physiology Institute and Sarah W. Stedman Nutrition and Metabolism Center, Duke University Medical Center, Durham, NC USA; 2 Department of Pharmacology & Cancer Biology, Duke University Medical Center, Durham, NC USA; 3 Division of Endocrinology, Metabolism, & Nutrition, Department of Medicine, Duke University Medical Center, Durham, NC USA

**Keywords:** Biochemistry, Metabolomics, Metabolic pathways, Metabolism

## Abstract

Protein acetylation is a fundamental regulatory mechanism occurring primarily on lysine amino acids. Here we report systematic in vivo characterization of cysteine S-acetylation as a widespread post-translational modification in mammalian tissues. By developing specialized sample preparation methods that preserve the labile thioester bond, we identified over 400 sites of cysteine acetylation in mouse liver, mirroring the abundance of lysine acetylation. Proteomic surveys across nine murine tissues revealed tissue-specific acetylation patterns that are enriched on metabolic enzymes in the cytoplasm. Cold exposure in mice triggers coordinated remodeling of the brown adipose tissue cysteine acetylome. Functional studies demonstrate that the acetylation of GAPDH Cys150 abolishes catalytic activity and correlates with nuclear enrichment, paralleling the known effects of S-nitrosylation on this enzyme. These findings establish cysteine acetylation as a widespread modification of metabolic proteins that responds to changes in cellular acetyl-CoA availability, fundamentally expanding the landscape of protein acetylation beyond lysine.

## Introduction

Protein post-translational modifications (PTMs) are fundamental to cellular regulation across all domains of life, vastly expanding the functional diversity of the proteome beyond what is encoded by the genome. These chemical modifications, which predominantly occur on amino acid side chains, enable dynamic regulation of protein structure, function, localization, and interactions. The advent of high-resolution mass spectrometry and sophisticated computational tools has revolutionized our ability to discover and characterize PTMs, revealing over 500 distinct modifications that play crucial roles in virtually every cellular process and human disease^1^.

Among the twenty canonical amino acids, cysteine stands out for its unique chemical reactivity under physiological conditions. The thiol group of cysteine, with its relatively low pKa compared to other nucleophilic amino acids, renders it highly reactive and versatile in biological systems. This reactivity underlies cysteine’s central role in enzyme catalysis, where it often serves as the nucleophilic active site residue in numerous enzyme families. Beyond catalysis, cysteine residues are frequent targets of post-translational modifications that regulate protein function. Well-characterized cysteine modifications include S-nitrosylation, which mediates nitric oxide signaling^2^, succination, which occurs under metabolic stress^3^, and S-palmitoylation, a reversible lipid modification that regulates protein trafficking and membrane association^4,5^. Additionally, cysteine modifications play critical roles in redox biology, serving as sensors and mediators of oxidative stress responses^6^.

The landscape of protein acylation has been particularly well-studied for lysine residues, where acetylation represents the prototypical short-chain acyl modification. Lysine acetylation was first discovered on histones, where it regulates chromatin structure and gene expression, but has since been found to modify thousands of proteins throughout the cell^7,8^. Importantly, lysine acetylation occurs through both enzymatic mechanisms, mediated by acetyltransferases and deacetylases, and non-enzymatic mechanisms, particularly in metabolically active compartments such as the mitochondrial matrix where acetyl-CoA concentrations are high and the pH is slightly elevated^9,10^. This paradigm of non-enzymatic acylation has been extended to other acyl-CoA species, demonstrating that the cellular acyl-CoA pool can spontaneously modify lysine residues with various acyl groups^11,12^.

Recent work has revealed an intriguing mechanistic link between cysteine and lysine modifications. James et al. demonstrated that non-enzymatic lysine acetylation can proceed through a cysteine intermediate, where acetyl-CoA first modifies a proximal cysteine residue via thioester formation, followed by intramolecular transfer of the acetyl group to a nearby lysine^13^. While these experiments were performed in vitro using synthetic peptides and isolated proteins, they raise the provocative possibility that cysteine residues might harbor short-chain acyl modifications in vivo. The chemical principles underlying this mechanism and the high reactivity of the cysteine thiol with acyl-CoA thioesters under physiological conditions suggest that direct cysteine acylation could be a widespread phenomenon^14^.

Despite the chemical feasibility of short-chain cysteine acylation, such modifications have remained undetected in biological systems. This contrasts sharply with long-chain cysteine acylation, particularly S-palmitoylation, which is well-established as a regulatory modification affecting thousands of proteins^15^. We thought that the absence of reported short-chain cysteine acylation likely reflects technical rather than biological limitations. The thioester bond connecting acyl groups to cysteine is inherently labile, being susceptible to hydrolysis at neutral pH, cleavage by reducing agents commonly used in proteomics workflows (such as DTT), and nucleophilic attack by other thiols^16,17^. These chemical properties would cause acyl cysteine modifications to be lost during standard sample preparation procedures, rendering them undetectable to conventional proteomic analyses.

Given the established reactivity of cysteine residues, the precedent of non-enzymatic protein acylation, and the mechanistic evidence for cysteine-acyl intermediates, we hypothesized that short-chain acyl modifications of cysteine exist in vivo but have evaded detection due to their chemical lability. To test this hypothesis, we developed specialized sample preparation protocols that preserve thioester-linked modifications while enabling comprehensive proteomic analysis. Focusing initially on acetylation as the prototypical short-chain acyl modification, we sought to identify and characterize a putative cysteine acetylome in mammalian tissues. Such modifications could represent a previously unrecognized layer of metabolic regulation, potentially linking cellular acyl-CoA pools to protein function through direct cysteine modification. Here we report the discovery and characterization of widespread cysteine S-acetylation from in vivo tissues, revealing a previously hidden landscape of protein regulation with implications for metabolism, cellular signaling, and our understanding of the relationship between protein acylation and cellular physiology.

## Results

### Identification and confirmation of cysteine S-acetylation

**Fig. 1:**
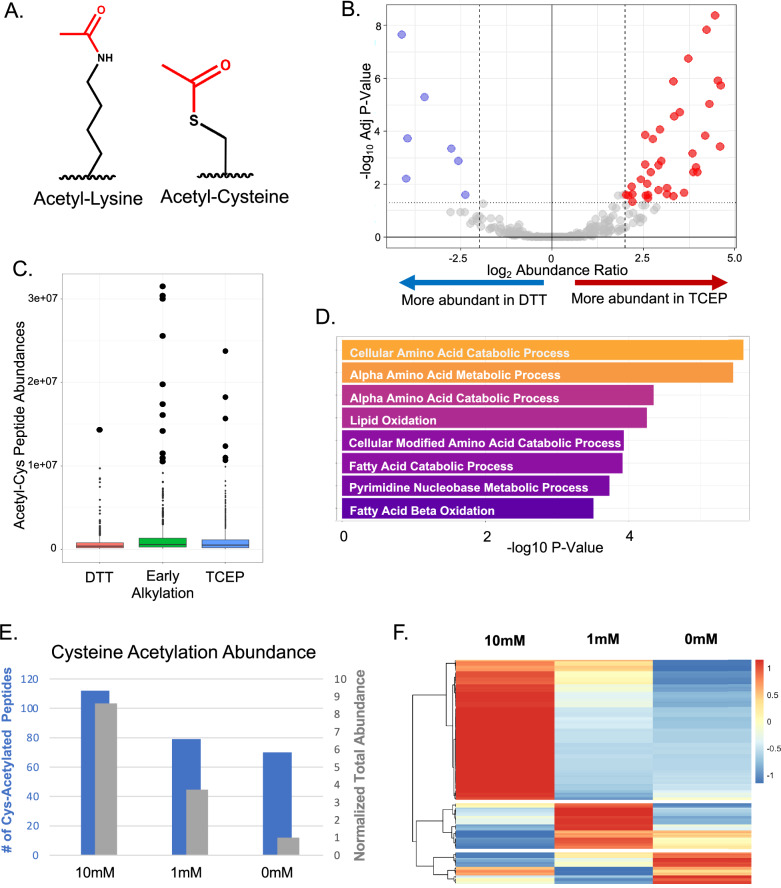
Identification of the cysteine acetylome by MS analysis and ablation of the cysteine acetylome by reduction with DTT. **A** Chemical structures of Acetyl-Lysine and Acetyl-Cysteine, showing the amine-linked acetyl modification on lysine and the thioester-linked acetyl modification on cysteine. **B** Volcano plot showing relative abundance (Log2 TCEP/DTT) vs statistical significance (-Log10 Adjusted P-value) of sites of cysteine acetylation in mouse liver tissue following reduction with TCEP compared to reduction with DTT. Samples were prepared using a modified S-trap protocol with pH maintained at 6.5-7.0. Cutoffs at 2-fold change and p < 0.05 were used to highlight sites that are more abundant with TCEP (red, *n* = 89% of significant sites) or DTT (blue). The ablation of cysteine acetylation signal by DTT confirms the thioester bond nature of the modification. **C** Distribution of peptide abundances of all cysteine acetylated peptides from initial preparation (without immediate alkylation, ~1 hour RT incubation) compared with early rapid alkylation preparation (immediate alkylation upon thawing). Peptides from initial prep are separated according to reducing agent (TCEP or DTT). Box plots show median, quartiles, and range. Abundances greater than 1×10^7 are shown with enlarged data points for emphasis. Early alkylation resulted in higher abundance of acetyl-cysteine peptides and more consistent identification. **D** Pathway overrepresentation analysis of cysteine acetylated proteins identified from mouse liver samples prepared using rapid early alkylation protocol (n = 218 acetyl-cysteine peptides out of 32,314 unique peptides). Overrepresentation analysis was performed against all proteins identified in the sample as background using Fisher’s exact test. Top eight enriched pathways shown with -log10 P-value. All pathways shown have p < 0.01 and FDR < 0.05. **E** Cysteine acetylation abundance in mouse liver lysate following incubation with 0, 1, or 10 mM acetyl-CoA for 1 hour at room temperature. Blue bars show number of unique cysteine-acetylated peptides identified; gray bars show normalized total abundance (summed intensity of acetyl-cysteine peptides normalized to total peptide signal). **F** Heatmap showing site-specific changes in cysteine acetylation abundance following acetyl-CoA treatment. Hierarchical clustering performed using correlation distance metric and average linkage. Color scale represents normalized abundance with red indicating increased acetylation and blue indicating decreased acetylation relative to control.

To identify whether acetyl-cysteine (Fig. 1A) was present in an in vivo setting, we performed label-free non-targeted mass spectrometry (MS) analysis of the mouse liver proteome from freshly harvested C57/Bl6J wild-type mouse liver samples without any enrichment. The inherent lability of an S-linked short-chain acyl PTM rendered standard protocols for preparing tissue samples for MS analysis unsuitable for these studies. The thioester bond connecting acyl modifications to the cysteine side chain is sensitive to temperature, pH, and reducing agents, and is susceptible to nucleophilic attack, all of which degrade short-chain cysteine acyl PTMs over time^16^.

To address these challenges, we developed a specialized MS sample preparation system that preserves the cysteine acetylome. Our optimized protocol incorporated several key modifications: First, we used tris(2-carboxyethyl)phosphine (TCEP) as the reducing agent, which reduces disulfide bonds while minimally reacting with thioester bonds, thus preserving acetyl-cysteine modifications compared to commonly used dithiothreitol (DTT)^18^. Second, sample pH was maintained at or below 7.0 throughout preparation using a modified protocol of the commercially available S-trap sample preparation system^19^, as higher pH accelerates thioester hydrolysis. Third, we streamlined the entire workflow to minimize processing time, achieving complete sample preparation in as little as 5 hours from processing frozen tissue to dried peptides ready for MS analysis. This rapid processing included immediate lysis of frozen pulverized tissue, protein extraction, tryptic digestion on S-trap columns, and peptide elution followed by speed vacuum drying.

Peptide samples were analyzed by reversed-phase nanoflow LC-MS/MS, using data-dependent acquisition on a Q Exactive Plus Orbitrap mass spectrometer. Raw data were searched against the UniProt mouse proteome database using both cysteine acetylation (+42.011 Da) and lysine acetylation as variable modifications. Initial searches revealed over 400 sites of cysteine side-chain acetylation in the mouse liver proteome. Out of 25,438 total unique peptides identified, 463 (1.8%) contained putative cysteine side-chain acetylation. These acetyl-cysteine-containing peptides represented 8.4% of the 5512 cysteine-containing peptides identified. For comparison, 384 peptides bearing lysine acetylation modifications were identified from these same samples, suggesting the cysteine acetylome is approximately as extensive as the lysine acetylome but has remained undetected due to incompatible sample preparation methods. Importantly, the majority of the 463 acetyl cysteine hits exhibited *local* FDR below 1% (PEP < 0.01), validating them as not being false positives in the 25,438 peptides identified at *global* FDR < 1% in unenriched samples^20^.

To confirm the chemical nature of the observed modification, we tested the reactivity of putative acetyl-cysteine by differentially treating samples with reducing agents and quantitatively monitoring the results. Parallel samples were prepared with either TCEP or DTT as the reducing agent. While TCEP selectively reduces disulfide bonds, DTT reduces both disulfide bonds and thioester bonds^17,21^, and therefore would be predicted to remove acetyl-cysteine modifications. The proteomic workflow was coupled with label-free quantitation to monitor the differential abundance of acetyl-cysteine peptides. If the observed modification was indeed thioester-linked acetyl-cysteine, DTT treatment would be expected to ablate the signal.

Comparing results from TCEP- and DTT-treated samples revealed a marked reduction of acetyl-cysteine signal in the DTT condition (Fig. 1B). Of the acetyl-cysteine peptides showing statistically significant changes (FDR-adj *p* < 0.05), 89% showed decreased abundance in the DTT condition. This DTT-labile behavior confirmed the presence of a thioester bond linking the acetyl modification to cysteine side chains. Combined with the observed mass shift of +42.011 Da matching an acetyl group localized to cysteine residues in MS/MS spectra, these results provided strong evidence that the identified PTM was indeed cysteine S-acetylation.

The lability of S-acetylcysteine extends beyond sensitivity to reducing agents. This modification likely forms when the deprotonated cysteine thiol attacks the thioester bond of acetyl-CoA^13^, creating a new thioester bond between the cysteine and acetyl moiety. This thioester remains susceptible to nucleophilic attack by other cysteine residues, potentially leading to acetyl transfer between cysteines. During cell lysis and sample preparation, the sudden exposure of many cysteine residues could enable widespread intermolecular acetyl transfer, scrambling the native acetylation pattern.

To test whether acetyl transfer affects the observed cysteine acetylome, we compared two alkylation strategies. In one approach, frozen liver lysates were immediately alkylated with iodoacetamide upon thawing to cap free cysteines. In the parallel approach, samples were incubated at room temperature for approximately 1 hour before alkylation. Delayed alkylation led to a markedly different cysteine acetylome profile (Supplemental Tables 1 and 2), with more diffuse and stochastic acetylation patterns consistent with extensive acetyl transfer. The delayed alkylation protocol identified roughly twice as many low-abundance acetyl-cysteine sites, but these showed poor quantitative reproducibility between replicates.

In contrast, immediate alkylation upon thawing yielded more consistent and reproducible results. While fewer unique acetyl-cysteine peptides were identified (218 peptides out of 32,314 total unique peptides), the abundance levels of these sites were higher and more reliably quantified (Fig. 1C). This represented a modification fraction of 4.7% of the 4649 cysteine-containing peptides identified. The lower fraction of total cysteine-containing peptides compared to our initial protocols (8.4%, described above) could reflect better preservation of other thioester-containing modifications, including long-chain acylations like palmitoylation, which would compete for ionization but were not included in our database searches.

The immediate alkylation protocol enabled reliable identification of likely high-abundance cysteine acetylation sites, as estimated by peptide spectral matches (PSMs). Top hits in the liver cysteine acetylome included metabolically relevant proteins: catalase, pancreatic triacylglycerol lipase, glutathione S-transferase, and glyceraldehyde-3-phosphate dehydrogenase (GAPDH) (Table 1). This improved consistency and quantitative reliability provided a more accurate picture of the physiological cysteine acetylome.

**Table 1:** Top cysteine acetylation sites identified in mouse liver proteome ranked by peptide spectral matches (PSMs)

# PSMs	Protein, Gene, Acetylated Residue
90	Pancreatic triacylglycerol lipase, Pnlip, Q6P8U6, [C]
55	60S ribosomal protein L4, Rpl4, Q9D8E6, [C3]
45	Peroxisomal coenzyme A diphosphatase NUDT7, Nudt7, Q99P30, [C5]
44	Catalase, Cat, P24270, [C393]
32	Imidazolonepropionate hydrolase, Uroc1, Q3UEL5, [C8]
26	Glutathione S-transferase A1, Gsta1, P13745, [C18]
20	Elongation factor 1-alpha 1, Eef1a1, P10126, [K/C411]
20	Fatty acid-binding protein, liver, Fabp1, P12710, [C69]
19	2’-5’-oligoadenylate synthase 2, Oas2, E9Q9A9, [C729]
18	Sorbitol dehydrogenase, Sord, Q64442, [K/C]
12	Glyceraldehyde-3-phosphate dehydrogenase, Gapdh, P16858, [K/C150]
11	Hemoglobin subunit beta-1, Hbb-b1, P02088, [K/C]
10	Glutathione S-transferase P 1, Gstp1, P19157, [K/C]

To understand the biological functions of the cysteine acetylated proteins, we performed pathway overrepresentation analysis comparing S-acetylated proteins against all proteins identified in our dataset. This analysis revealed a strong metabolic signature in the cysteine acetylome. Cellular Amino Acid Catabolic Process and Alpha Amino Acid Metabolic Process were the most significantly enriched pathways (p < 0.01, FDR < 0.05) (Fig. 1D). Additional enriched pathways included Lipid Oxidation, Fatty Acid Catabolism, and Fatty Acid Beta-Oxidation. This metabolic enrichment aligned with the identification of key metabolic enzymes among the top acetylated proteins, including glutamate dehydrogenase, mitochondrial trifunctional protein, and GAPDH (Table 1). These results definitively demonstrate that cysteine S-acetylation occurs in mouse liver tissue in vivo and is enriched on metabolic proteins.

### Acetyl-CoA serves as the acyl donor for cysteine acetylation

Having established that cysteine S-acetylation occurs in vivo, we next investigated the source of the acetyl group. Given that acetyl-CoA is the canonical acetyl donor for lysine acetylation and other cellular acetylation reactions^1,12^, we hypothesized that acetyl-CoA would similarly serve as the acetyl donor for cysteine acetylation. To test this, we incubated mouse liver lysate with increasing concentrations of acetyl-CoA and monitored changes in the cysteine acetylome by quantitative mass spectrometry.

Fresh mouse liver lysate was prepared and immediately treated with 0, 1, or 10 mM acetyl-CoA for 1 hour at room temperature, followed by alkylation to halt further acetylation. These concentrations were chosen to span physiological (1 mM) to supraphysiological (10 mM) levels of acetyl-CoA. As predicted, acetyl-CoA treatment led to robust, concentration-dependent increases in the number of cysteine acetylated peptides identified (Fig. 1E). Treatment with 1 mM acetyl-CoA resulted in a 3.7-fold increase in the summed intensity of cysteine acetylated peptides (normalized to total peptide signal), while the number of unique acetylated peptides increased by only 13%. This suggested to us that physiological concentrations of acetyl-CoA primarily increase the stoichiometry of acetylation at existing sites rather than creating new sites of modification.

In contrast, treatment with 10 mM acetyl-CoA had more dramatic effects, increasing the summed intensity of cysteine acetylated peptides 8.6-fold while expanding the number of unique acetylated sites by 60%. This concentration-dependent expansion of the cysteine acetylome suggests that supraphysiological acetyl-CoA concentrations acetylated previously unmodified cysteines, possibly reflecting differences in cysteine reactivity or accessibility.

We found acetyl-CoA-induced increases in cysteine acetylation were highly site-specific (Fig. 1F, Supplemental Table 3). Different proteins and even different cysteine residues within the same protein showed distinct dose-response relationships. Catalase showed rapid response kinetics, with Cys393 and Cys460 showing near-maximal acetylation increases at 1 mM acetyl-CoA that plateaued at higher concentrations. This saturable response suggests these cysteines may be particularly reactive or accessible to acetyl-CoA.

In contrast, glutamate dehydrogenase (GDH) displayed linear dose-response kinetics. Acetylation of GDH Cys112 increased 4.5-fold with 1 mM acetyl-CoA and 36-fold with 10 mM acetyl-CoA, suggesting this site remains responsive across a broad range of acetyl-CoA concentrations. Such linear responses may indicate sites where acetylation is limited by acetyl-CoA availability rather than cysteine reactivity.

Most strikingly, GAPDH (S4R1W1 isoform) showed a threshold response, with no detectable increase in active site acetylation at 1 mM acetyl-CoA but a 15-fold increase at 10 mM. This all-or-nothing behavior suggests that certain cysteines may require high local acetyl-CoA concentrations to overcome kinetic or thermodynamic barriers to acetylation, possibly due to protein conformation or microenvironment effects.

### Subcellular Distribution of the Cysteine Acetylome

Because lysine acetylation has strong sub-cellular distribution patterns^7,8^, we investigated the subcellular distribution of cysteine acetylation. The strong overrepresentation of metabolic pathways in the cysteine acetylome suggested the possibility that this modification could be enriched in metabolically active compartments of the cell. To test this, freshly harvested mouse liver samples were fractionated using a rapid differential centrifugation protocol into three fractions based on their sedimentation properties: a heavy membrane fraction (containing mitochondria, nuclei, and peroxisomes), a light membrane fraction (containing endoplasmic reticulum, Golgi apparatus, and microsomes), and a supernatant fraction (containing cytoplasmic proteins). To preserve the cysteine acetylome signature during the subcellular fractionation process, protocols were optimized to control temperature and pH. Time was also a critical factor, with fractionation streamlined to minimize degradation of the labile thioester bonds. Each fraction was then prepared for MS analysis using the protocols described above.

**Fig. 2:**
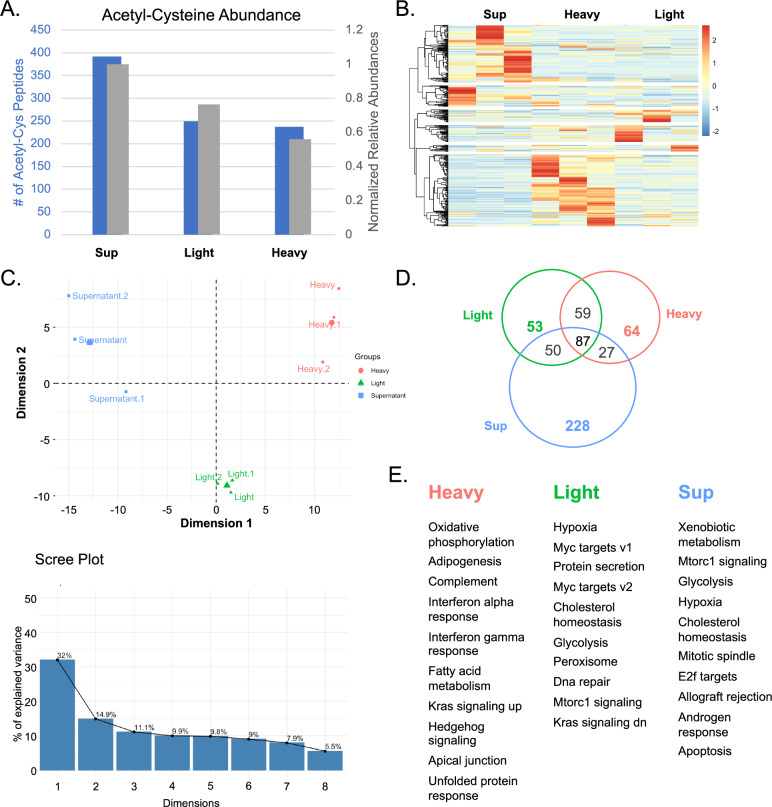
Analysis of the cysteine acetylome following subcellular fractionation of mouse liver into heavy membrane, light membrane, and supernatant fractions. **A** The summed intensity of cysteine acetylated peptides normalized to total peptide signal (gray bars), and the number of unique S-acetylated peptides identified (blue bars) for each subcellular compartment. Mouse liver was fractionated by differential centrifugation at 70 g, 700 g, and 7,000 g. Supernatant fraction showed highest levels of cysteine acetylation with 392 unique peptides identified. **B** Heatmap showing distribution of cysteine acetylome in each subcellular compartment. Hierarchical clustering was performed using the correlation distance metric. Color scale represents normalized relative abundance with red indicating high abundance and blue indicating low abundance. Each row represents a unique acetyl-cysteine site. **C** Principal Component Analysis (PCA) of the cysteine acetylome proteins present in heavy membrane (containing mitochondria and nuclei), light membrane (containing ER and golgi), and supernatant (cytoplasmic) subcellular fractions. PC1 and PC2 account for 32% and 14.9% of variance, respectively. Scree plot (inset) showing the relative contribution of each of the first 8 components. **D** Venn diagram showing the extent of overlap of the cysteine acetylome between subcellular compartments as measured by shared sites of cysteine acetylation. Numbers indicate unique acetyl-cysteine peptides identified in each fraction or combination of fractions. Only 87 peptides (22%) were shared between all three compartments. **E** Pathway overrepresentation analysis of the cysteine acetylome for each compartment. Showing top 10 pathways for each fraction. Pathway overrepresentation analysis was performed using cysteine acetylated genes for each compartment against the list of all genes identified in that compartment as background.

Unexpectedly, the cytoplasmic supernatant fraction had the highest levels of cysteine acetylation, measured both by the number of sites identified and by the summed intensity of cysteine acetylated peptides normalized to total peptide signal. The heavy membrane fraction, despite containing metabolically active organelles such as mitochondria, showed the lowest levels of overall cysteine acetylation (Fig. 2A). This cytoplasmic enrichment contrasts with lysine acetylation, which is often enriched in mitochondria where acetyl-CoA concentrations are highest.

In addition to differences in overall levels of acetylation, each subcellular compartment showed distinct patterns of cysteine acetylation (Fig. 2B). Principal component analysis (PCA) on proteins (Fig. 2C) or peptides (Supplementary Figures 2, 3) revealed a clear separation of the cysteine acetylome for each subcellular compartment, indicating that the pattern of cysteine acetylation on proteins was distinct between compartments. The first and second components of the protein PCA accounted for 32% and ~15% of the observed variance, respectively (Fig. 2C). Notably, the peptide PCA plot, whose abundances have been normalized to their parent proteins, shows that even after accounting for underlying protein abundance differences, group separation strongly suggests bona fide differences in acetylation levels rather than consequences of different underlying protein abundances.

In total, 392 acetyl-cysteine-containing peptides were identified in the cytoplasmic supernatant fraction, 249 in the light membrane fraction (ER/Golgi-enriched), and 237 in the heavy membrane fraction (mitochondria/nuclei-enriched). Strikingly, only 87 peptides (approximately 22% of all identified sites) were shared between all three compartments (Fig. 2D), suggesting that cysteine acetylation may have compartment-specific functions. Subcellular fractionation resulted in more acetyl-cysteine peptides being identified compared to unfractionated samples, likely due to the reduced lysate complexity, allowing identification of lower abundance modifications that would be masked in whole tissue.

Overrepresentation analysis was performed on the proteins identified in each subcellular fraction to identify pathways enriched with cysteine acetylation. The top ten pathways from each fraction showed biological processes known to occur in the organelles in that fraction, confirming successful fractionation (Fig. 2E). Notably, metabolic pathways and their regulatory counterparts were overrepresented in all fractions.

### Tissue-specific patterns of cysteine acetylation across murine organs

To determine whether cysteine acetylation is a general phenomenon or has tissue-specific patterns, we performed a comprehensive survey of the cysteine acetylome across nine murine tissues. Brown adipose tissue (BAT), brain, heart, kidney, liver, lung, pancreas, skeletal muscle, and spleen were harvested from male, three-month-old C57/Bl6J wild-type mice (n = 3 biological replicates per tissue). All tissues were processed using our optimized protocol with immediate alkylation to preserve native acetylation patterns.

For this multi-tissue survey, we adopted a streamlined analytical approach focused on identifying the most abundant and reproducible sites of cysteine acetylation. While our initial liver characterization employed up to 18 LC-MS/MS runs for deep proteome coverage, resource constraints limited this survey to three runs per tissue (one per biological replicate). This strategy prioritized the detection of high-abundance acetylation sites, which we reasoned are most likely to have biological significance. To assess technical reproducibility and enable cross-tissue normalization, we created a quality control (QC) pool by combining equal amounts of peptide digest from all tissues. This QC pool was analyzed in five technical replicate injections interspersed throughout the sample queue, providing both a technical performance metric and an empirical average of the combined tissue proteomes for chromatographic alignment.

**Fig. 3:**
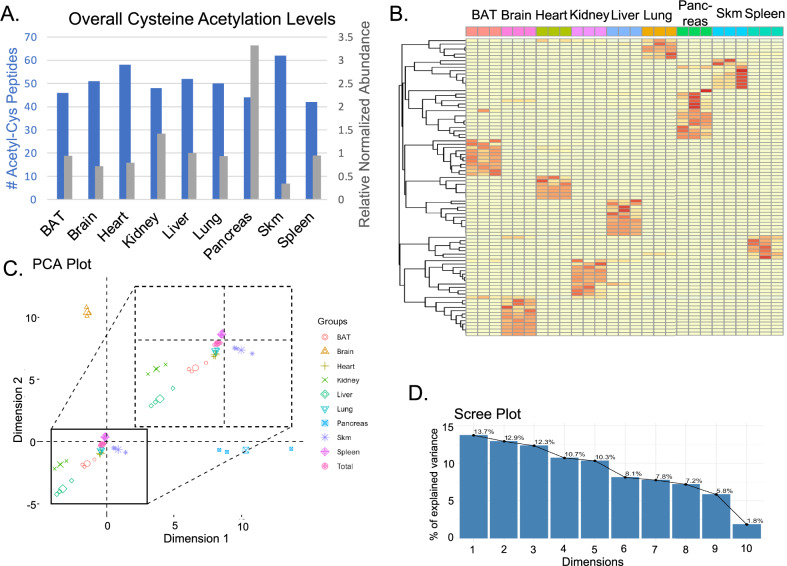
Multi-tissue survey of the murine cysteine acetylome. **A** The summed intensity of cysteine acetylated peptides, normalized to total peptide signal (gray bars) and the number of unique S-acetylated peptides identified (blue bars) for each tissue type. Tissues harvested from male, 3-month-old C57/Bl6J mice (*n* = 3 biological replicates per tissue). Pancreas showed highest normalized abundance (3.3x liver levels), while skeletal muscle showed lowest (0.3x liver levels). **B** Heatmap showing the distribution of the cysteine acetylome for different tissues. Hierarchical clustering performed using correlation distance metric. Each row represents a unique acetyl-cysteine site, columns represent individual tissue samples. Color scale shows normalized abundance. **C** PCA of the cysteine acetylome across all tissue types studied. Pooled QC sample labeled as “Total” (created by combining equal amounts of each tissue peptide digest, *n* = 5 technical replicates). Brain, skeletal muscle (Skm), and heart show greatest separation from other tissues. PC1 and PC2 account for 13.7% and 12.9% of variance, respectively. **D** Scree plot showing the relative contribution of each of the first 10 principal components to the total variance in the multi-tissue cysteine acetylome dataset.

The multi-tissue analysis revealed striking tissue-specific patterns of cysteine acetylation. Each tissue displayed a unique acetylome signature, both in terms of which proteins were acetylated (Fig. 3A) and the overall extent of acetylation (Fig. 3B). PCA clearly separated tissues based on their cysteine acetylation patterns, with the first two components explaining 26.6% of the total variance (PC1: 13.7%, PC2: 12.9%) (Fig. 3C and D). Notably, brain, skeletal muscle, and heart showed the greatest separation from other tissues, suggesting these organs harbor particularly distinct cysteine acetylomes. In contrast, kidney, lung, and spleen clustered near the QC pool average (‘Total’), indicating their acetylation patterns more closely resemble the pan-tissue average (Fig. 3C).

The technical reproducibility of our measurements varied by tissue. For most tissues, biological variability exceeded technical variability, as evidenced by greater dispersion among biological replicates than among QC pool injections. However, heart samples showed remarkably low inter-sample variability, comparable to technical replicates, suggesting the cardiac cysteine acetylome may be under tight regulatory control or less susceptible to individual variation.

Analysis of specific acetylated proteins revealed both tissue-specific and broadly distributed modifications (Table 2). Each tissue exhibited a characteristic set of highly acetylated proteins that likely reflect tissue-specific metabolic demands and functional specializations. For example, skeletal muscle showed prominent acetylation of actin, consistent with the importance of cytoskeletal regulation in contractile tissue. The liver acetylome was enriched for metabolic enzymes including GAPDH, aligning with its central role in metabolism. Surprisingly, pancreatic triacylglycerol lipase was found acetylated not only in pancreas but also in BAT, liver, and spleen, suggesting this modification may regulate lipase function across multiple tissues involved in lipid metabolism. Among all proteins surveyed, only ribosomal protein RPL4 showed consistent acetylation across all nine tissues, indicating that cysteine acetylation of the translational machinery may be a conserved phenomenon.

**Table 2:** Tissue-specific distribution of highly abundant cysteine acetylation sites across nine murine organs

Liver	
# PSMs	Protein, Gene, Acetylated Residue(s)
9	Large ribosomal subunit protein uL4, Rpl4, [C3]
8	Pancreatic triacylglycerol lipase, Pnlip, [C313(33.3); C316(33.3); C321(33.3)]
7	Peroxisomal coenzyme A diphosphatase NUDT7, Nudt7, [C5]
6	urocanate hydratase, Uroc1, [C8]
5	Alpha globin 1, Hba-a1, [C105] Hemoglobin subunit alpha, Hba, [C105]
5	Glyceraldehyde-3-phosphate dehydrogenase, Gm3839, [C150(50); C154(50)]
5	RIKEN cDNA 1700014D04 gene, 1700014D04Rik, [K358(50); C359(50); C370(50); C371(50)]
4	Acetyl-CoA acetyltransferase, mitochondrial, Acat1, [C116]
4	Splicing regulator SDE2, Sde2, [C403; K411; C412]
3	2’-5’-oligoadenylate synthase 2, Oas2, [C729]
3	Leucine rich repeat containing 47, Lrrc47, [K495(50); C498(50); C513(100); K514(100)]

### Dynamic regulation of the cysteine acetylome during cold-induced thermogenesis

To investigate whether cysteine acetylation responds to physiological stimuli, we examined brown adipose tissue (BAT) during cold-induced thermogenic activation. BAT undergoes dramatic metabolic reprogramming upon cold exposure, increasing glucose uptake, lipid oxidation, and mitochondrial uncoupling to generate heat^22,23^. This metabolic shift is accompanied by elevated acetyl-CoA production, making BAT an ideal model to test whether physiological changes in metabolism alter the cysteine acetylome.

**Fig. 4:**
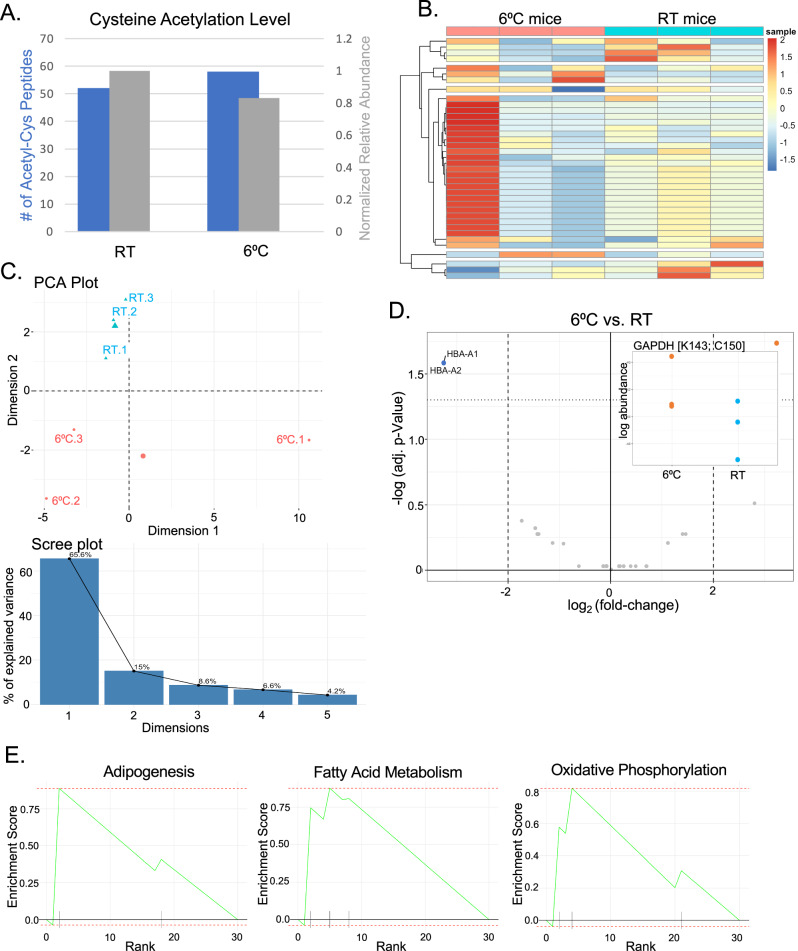
Cysteine acetylome in BAT from mice acclimated to 6 °C compared to mice housed at RT. **A** Overall abundance of the cysteine acetylome in BAT at room temperature (RT, 22 °C) and 6 °C. Gray bars show the summed intensity of cysteine acetylated peptides, normalized to total peptide signal, and blue bars show the number of unique S-acetylated peptides identified. Age-matched mice were cold-acclimated by housing at 6 °C for 4 weeks (n = 3 per group). **B** Heatmap showing differences in the distribution of the cysteine acetylome in BAT from mice housed at RT vs mice housed at 6 °C. Hierarchical clustering performed using correlation distance metric. Each row represents a unique acetyl-cysteine site. Color scale represents normalized relative abundance. **C** PCA of the cysteine acetylome in BAT from RT and 6 °C mice. PC1 accounts for 65.6% of variance and clearly separates the two temperature conditions. Scree plot (inset) showing the relative contribution of each of the first 5 components. **D** Volcano plot showing fold-change in abundance of cysteine acetylation at sites acetylated in both the RT and 6 °C conditions. Cutoffs at 2× fold change and p < 0.05. GAPDH active site acetylation (K143; C150) and hemoglobin A (HBA) showed statistically significant changes. Points colored by temperature condition where peptide was more abundant. **E** Gene Set Enrichment Analysis (GSEA) plots showing enrichment for Adipogenesis, Fatty Acid Metabolism, and Oxidative Phosphorylation pathways in the cysteine acetylome of 6 °C acclimated mice compared to RT housed mice. Analysis performed using fgsea with 10,000 permutations.

Male C57/Bl6J mice were housed either at room temperature (22 °C) or cold-acclimated at 6 °C for 4 weeks to achieve stable thermogenic activation (n = 3 per group). BAT was harvested and immediately processed using our optimized protocol to preserve native cysteine acetylation patterns. Comparative proteomics revealed that while the overall abundance of cysteine acetylation remained similar between conditions (Fig. 4A), cold exposure induced a substantial reorganization of the cysteine acetylome.

The redistribution of cysteine acetylation was evident at both global and individual protein levels. Hierarchical clustering revealed distinct acetylation patterns between room temperature and cold-exposed samples (Fig. 4B). PCA provided striking separation of the two conditions, with PC1 alone accounting for 65.6% of the total variance (Fig. 4C). This remarkable separation along a single component suggests that cold exposure induces a coordinated shift in the cysteine acetylome rather than random changes across multiple proteins.

Gene set enrichment analysis (GSEA) revealed significant enrichment of three key metabolic pathways in cold-adapted BAT: Adipogenesis, Fatty Acid Metabolism, and Oxidative Phosphorylation (Fig. 4E). The enrichment of these pathways aligns with the known metabolic adaptations to cold, suggesting cysteine acetylation participates in the metabolic rewiring required for thermogenesis.

Among individual proteins showing significant changes, GAPDH displayed markedly increased active site acetylation (K143 and C150) in cold-exposed BAT (Fig. 4D). This finding was particularly intriguing given that GAPDH was also identified as one of the most highly acetylated proteins in our initial liver proteome survey (Table 1). The convergent identification of GAPDH acetylation across multiple tissues and conditions prompted detailed functional characterization of this modification.

### Cysteine acetylation inhibits GAPDH catalytic activity

**Fig. 5:**
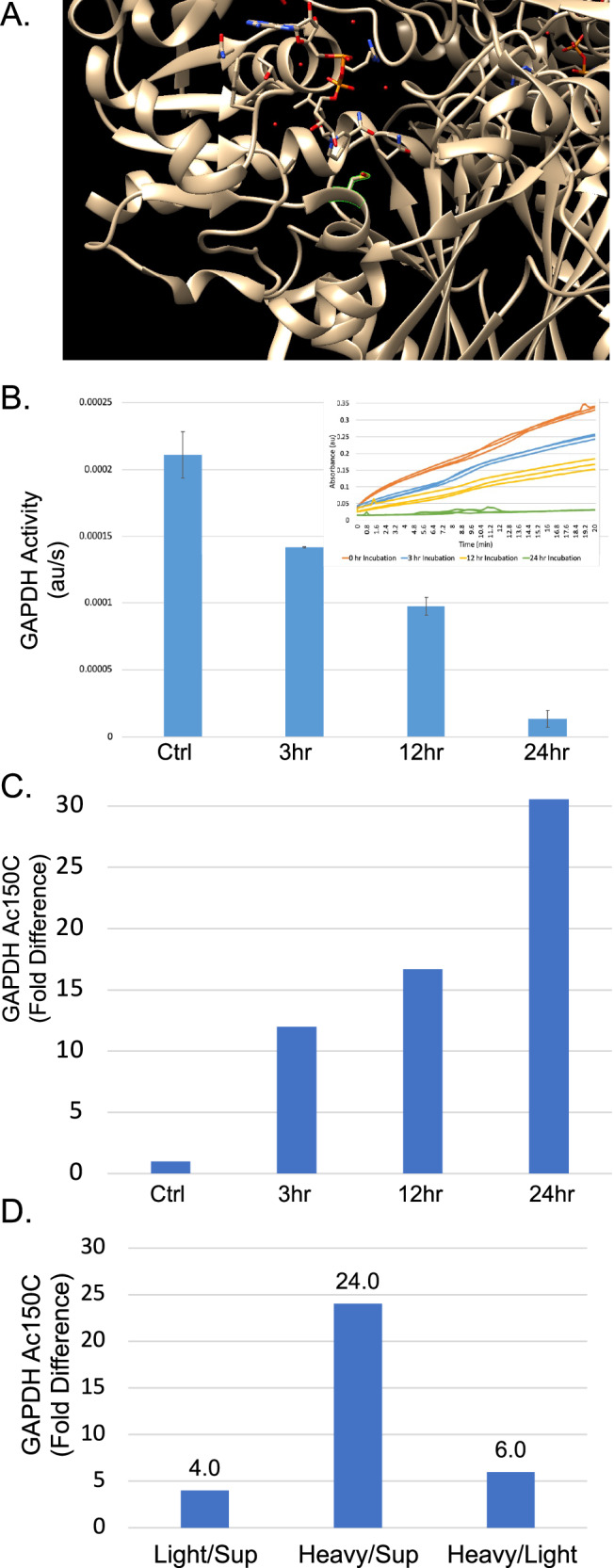
GAPDH acetylation and activity. **A** Activity of GAPDH as determined by in vitro enzymatic assay upon increasing levels of cysteine acetylation. Purified rabbit GAPDH (1 mg/mL) was incubated with 10 mM acetyl-CoA at 4 °C for 0, 3, 12, and 24 hours. All samples were kept at 4 °C for total of 24 hours to control for temperature effects. Activity measured using a commercial GAPDH activity assay kit. Error bars represent SEM (*n* = 3). Inset shows raw kinetic traces for each condition. **B** Extent of GAPDH active site acetylation upon increasing length of incubation with 10 mM acetyl-CoA. Fold change calculated from MS analysis of acetylation at Cys150 and Cys154 relative to control (0 hour incubation). After 24 hours, active site acetylation increased ~30-fold while activity decreased 90%. **C** Active site structure of GAPDH (PDB structure shown) with Cys150 highlighted in red. This catalytic cysteine is essential for enzymatic activity and is the primary site of acetylation. **D** Relative fraction of GAPDH that is cysteine acetylated at the active site in different subcellular compartments from mouse liver fractionation. Values shown as fold difference between compartments. Heavy membrane fraction (containing nuclei) showed 28x higher acetylation than supernatant and 18x higher than light membrane fraction. This enrichment was driven by the S4R1W1 isoform (A156S variant) which was consistently acetylated and localized to the heavy membrane fraction.

The murine GAPDH cysteine acetylome revealed modification of Cys150, a critical active site residue essential for catalytic activity (Fig. 5A). This cysteine is remarkably versatile, serving as a target for multiple PTMs including S-nitrosylation^24^, ADP-ribosylation^25,26^, and succination^3^. Notably, S-nitrosylation of Cys150 not only inhibits glycolytic activity but also triggers GAPDH nuclear translocation, where it participates in transcriptional regulation, DNA repair, and apoptosis^27–29^.

To determine whether cysteine acetylation similarly affects GAPDH function, we examined the relationship between acetylation and enzymatic activity. Purified rabbit GAPDH was incubated with 10 mM acetyl-CoA at 4 °C for varying durations (0, 3, 12, or 24 h), with all samples maintained at 4 °C for 24 hours total to control for temperature effects. Following incubation, samples were divided for parallel analysis of acetylation status by mass spectrometry and enzymatic activity. Time-dependent acetylation of GAPDH active site cysteines correlated inversely with catalytic activity. After 24 hours of acetyl-CoA exposure, enzymatic activity decreased by 90% (Fig. 5B), while active site acetylation increased approximately 30-fold (Fig. 5C). The near-complete loss of activity upon extensive acetylation confirms that modification of the catalytic cysteines, particularly Cys150, abolishes GAPDH’s enzymatic function.

### An acetylated GAPDH isoform localizes to nuclear-enriched fractions

Given the parallels between cysteine acetylation and nitrosylation in inhibiting GAPDH activity, we investigated whether acetylation might similarly influence subcellular localization. Analysis of our subcellular fractionation data revealed an unexpected finding: a minor GAPDH isoform (S4R1W1) showed constitutive active site acetylation and distinct subcellular distribution.

The S4R1W1 isoform differs from canonical GAPDH by a single amino acid substitution (A156S). Remarkably, across all proteomic datasets, every active site peptide from S4R1W1 was acetylated, except when treated with DTT to cleave thioesters. In contrast, the canonical isoform showed variable, typically low acetylation. This constitutive modification of S4R1W1 suggests it may represent a dedicated non-glycolytic form of GAPDH.

Supporting this hypothesis, S4R1W1 preferentially localized to the heavy membrane fraction containing nuclei and mitochondria. While total GAPDH was most abundant in the cytoplasmic fraction, the proportion bearing active site acetylation was 28-fold higher in the heavy membrane fraction (Fig. 5D). This enrichment was driven entirely by S4R1W1, where all S4R1W1 peptides in the heavy membrane fraction were acetylated, whereas canonical GAPDH peptides from this fraction showed no acetylation. The nuclear enrichment of acetylated S4R1W1 parallels the behavior of nitrosylated GAPDH, suggesting cysteine acetylation may serve as an alternative mechanism for redirecting GAPDH from glycolysis to nuclear functions.

## Discussion

The discovery of widespread cysteine S-acetylation fundamentally expands our understanding of protein post-translational modifications and reveals a previously hidden layer of cellular regulation. Despite the well-established reactivity of cysteine residues and extensive characterization of lysine acetylation, short-chain acylation of cysteine has remained undetected in mammalian tissues until now. Our findings demonstrate that cysteine acetylation is not merely a chemical curiosity but a physiologically relevant modification that rivals lysine acetylation in prevalence and exhibits tissue-specific patterns, subcellular localization, and dynamic regulation in response to metabolic stimuli.

The failure to previously detect cysteine acetylation reflects technical limitations rather than biology. The thioester bond linking acetyl groups to cysteine is inherently labile, susceptible to cleavage by commonly used reducing agents (particularly DTT), hydrolysis at neutral or basic pH, and nucleophilic attack by other thiols^16,18^. Standard proteomic workflows, optimized for stable modifications, inadvertently destroy cysteine acetylation. Our success required three critical innovations: substituting TCEP for DTT to preserve thioesters while reducing disulfides, maintaining acidic pH throughout sample preparation, and implementing immediate alkylation upon tissue lysis to prevent acetyl transfer between cysteines. The technical challenges extend beyond sample preparation. Unlike lysine acetylation, no antibodies recognize acetyl-cysteine, precluding enrichment strategies that have been instrumental in characterizing other PTMs. This forced us to detect cysteine acetylation within whole proteome analyses, limiting sensitivity and likely causing us to underestimate the true extent of this modification. Previous studies of cysteine acylation may have unknowingly detected short-chain modifications but, focusing exclusively on palmitoylation, attributed unidentified acyl-cysteine sites to technical artifacts^15,30^.

Our data support both enzymatic and non-enzymatic mechanisms for cysteine acetylation, with the balance likely depending on cellular context and acetyl-CoA availability. The concentration-dependent response to acetyl-CoA treatment, particularly the dramatic expansion of the acetylome at supraphysiological concentrations, indicates that non-enzymatic acetylation can occur. At physiological pH, the cysteine thiol (pKa ~8) is substantially more nucleophilic than the lysine amine (pKa ~10.5), making non-enzymatic acetylation of cysteine thermodynamically and kinetically favored^31^. However, the striking site specificity observed with some cysteines showing saturable acetylation at low acetyl-CoA concentrations while others require high concentrations or remain unmodified argues against purely stochastic modification. This specificity likely arises from multiple factors. Local protein microenvironment can shift cysteine pKa by several units, dramatically affecting reactivity^31^. Protein conformation controls solvent accessibility, while nearby residues may facilitate or hinder acetyl-CoA binding. The distinct dose-response curves for different sites (saturable for catalase, linear for GDH, threshold for GAPDH) suggest that cells may use acetyl-CoA concentration to regulate which proteins become acetylated, creating a hierarchical response to metabolic state. However, it is worth noting that the acetyl-CoA concentrations used (1-10 mM) in vitro represent approximately 10-100 fold molar excess over total cysteine residues in GAPDH, conditions that favor non-enzymatic acetylation. Future studies employing lower acetyl-CoA:cysteine ratios would help establish the threshold for physiologically relevant acetylation and distinguish high-affinity sites from those requiring elevated acetyl-CoA.

The predominant cytoplasmic localization of cysteine acetylation was initially unexpected given the metabolic pathway enrichment first observed (Fig. 1) and the fact that lysine acetylation is often enriched in mitochondria where acetyl-CoA production is highest. This compartmentalization suggests that cysteine acetylation may serve functions beyond direct metabolic regulation. The cytoplasmic enrichment could reflect several non-exclusive possibilities: protection of reactive cysteines from oxidation or unwanted disulfide formation, regulation of protein-protein interactions, or modulation of enzyme activities in response to cytoplasmic acetyl-CoA levels. Given the higher pKa of lysine compared to cysteine (~10.5 vs. ~8), the elevated pH of the mitochondrial matrix (~8.0 vs. ~7.2 in the cytosol) may lessen cysteine’s deprotonation advantage, thereby diminishing the nucleophilic superiority it exhibits over lysine under cytosolic conditions. In addition, acetylcysteine would presumably be more prone to hydrolysis at the elevated pH of the mitochondrial matrix. Further, the lower acetylation levels in the light membrane fraction may reflect the oxidizing environment of the ER, where cysteine residues preferentially form disulfides rather than undergo acetylation.

The tissue-specific patterns further support functional specialization. Metabolically active tissues (pancreas, liver, BAT) showed high acetylation abundance, consistent with acetyl-CoA availability driving modification. However, the distinct acetylated protein profiles in each tissue suggest that cysteine acetylation is not merely a passive reflection of metabolism but actively shapes tissue-specific functions. The identification of constitutively acetylated proteins (like RPL4 across all tissues) versus tissue-specific targets implies both housekeeping and specialized regulatory roles.

Our detailed characterization of GAPDH acetylation provides a paradigm for understanding how cysteine acetylation impacts protein function. Acetylation of the catalytic Cys150 abolishes enzymatic activity, similar to other modifications of this residue including nitrosylation and succination^3,24^. However, the discovery of the constitutively acetylated S4R1W1 isoform adds a new dimension. This minor isoform, differing by a single amino acid, appears dedicated to non-glycolytic functions, maintaining permanent acetylation and localizing to nuclear-enriched fractions. The parallel between acetyl-cysteine and nitrosyl-cysteine in redirecting GAPDH from metabolism to nuclear functions suggests these modifications may serve overlapping roles. While nitrosylation typically requires specific stimuli (NO production), constitutive acetylation of S4R1W1 could provide a steady-state pool of GAPDH available for moonlighting functions. This raises the intriguing possibility that cells use different cysteine modifications to fine-tune the balance between metabolic and non-metabolic functions of multifunctional proteins.

The enrichment of metabolic proteins in the cysteine acetylome, combined with acetyl-CoA-dependent modification, positions cysteine acetylation as a potential metabolic sensor. Unlike lysine acetylation, which often requires enzymatic machinery, cysteine acetylation could provide rapid, reversible response to fluctuating acetyl-CoA levels. The cold-induced reorganization of the BAT acetylome without changes in total acetylation demonstrates that this modification can be dynamically regulated rather than simply reflecting acetyl-CoA abundance. Several features make cysteine acetylation well-suited for metabolic regulation. The modification is readily reversible through thioester hydrolysis or enzymatic deacetylation. It can directly inhibit enzyme activity when occurring at catalytic cysteines. The hierarchical acetylation of different sites based on acetyl-CoA concentration could allow graded responses to metabolic state. These properties suggest cysteine acetylation may complement other metabolic PTMs, providing rapid fine-tuning of enzyme activities.

However, one major caveat to this speculation is the increased variability in cold-exposed mice. This likely reflects individual differences in the extent of thermogenic activation. Future studies employing shorter time courses (e.g., 1, 7, 14, and 28 days of cold exposure) would help distinguish acute responses from adaptive changes and potentially identify time points with more uniform acetylation patterns. Additionally, measuring thermogenic markers (UCP1 expression, core body temperature) in individual mice would allow correlation of acetylome changes with physiological adaptation state.

The discovery of cysteine acetylation has broad implications for understanding protein regulation and cellular metabolism. First, it expands the landscape of protein acetylation beyond lysine, potentially doubling the number of acetylation sites in the proteome. Second, it provides a new mechanism for metabolic regulation that directly links acetyl-CoA availability to protein function. Third, it offers new perspectives on multifunctional proteins like GAPDH, where cysteine modifications may switch between distinct functional states.

Technical advances will be crucial for progress. Development of antibodies or chemical probes specific for acetyl-cysteine would enable enrichment strategies and cellular imaging. For example, development of click-chemistry approaches using alkynyl acetyl-CoA analogs would provide valuable orthogonal validation and enable spatial visualization of cysteine acetylation in cells. Such tools would complement our MS-based discovery approach and facilitate mechanistic studies of individual acetylated proteins. Further, methods to stabilize or specifically detect other short-chain acyl modifications of cysteine could reveal additional layers of regulation. Alternatively, alternative cellular thioesters such as acetyl-GSH or related could serve as acetyl donors for cysteine acetylation, which would help distinguish specific acetyl-CoA effects from general thioester reactivity. Finally, future studies employing ^13^C-glucose tracers will be essential to quantify the metabolic impact of cysteine acetylation and determine whether this modification redirects metabolic flux, particularly under conditions of varying acetyl-CoA availability.

In conclusion, cysteine S-acetylation represents a widespread PTM that has remained hidden due to technical challenges rather than biological absence. Its discovery opens new avenues for understanding metabolic regulation and the interplay between different PTMs. Our findings add to an emerging picture of short-chain acylations on cysteine from cognate acyl-CoAs. For example, the discovery of S-lactoylation of cysteine residues demonstrated that lactyl-CoA can modify cysteines through a similar thioester mechanism^32^. Together with our identification of widespread cysteine acetylation, these findings suggest that cysteine may be subject to diverse acyl modifications derived from metabolic acyl-CoA species, paralleling the extensive acylation landscape known for lysine residues^1^. As we study this modification further, cysteine acetylation is poised to emerge as a key player in cellular regulation, complementing and interacting with the established landscape of protein modifications.

## Methods

### Animals

Ethical approval was obtained from the Institutional Animal Care and Use Committee (IACUC) at Duke University. The approved protocol is registered as Protocol Registry Number A035-23-02, titled “Modifications of Mouse Genes for Metabolic Research”. All animal experiments and procedures were conducted in strict accordance with the approved IACUC protocol and adhered to the guidelines and regulations set forth by the Animal Welfare Act and the Guide for the Care and Use of Laboratory Animals. All mice used in this study were wild type C57/Bl6J. Mice were housed in 12:12 light dark cycle facility and fed on standard chow. All mice used in this study were euthanized by CO_2_ exposure maintained for at least 3 minutes after respiratory arrest followed by decapitation or removal of vital organs.

### Tissue Collection and Preparation

Mice were euthanized as described above, and tissues were immediately removed and flash frozen by clamping with pliers pre-chilled in liquid nitrogen. Frozen tissues were stored at −80 °C until further processing. Tissues were pulverized using a mortar and pestle chilled in liquid nitrogen and stored at −80 °C until use. Pulverized tissue was weighed out into an Eppendorf tube chilled in liquid nitrogen to prevent thawing during the weighing process.

### Tissue Lysis and Protein Extraction

Tissue powder was thawed by adding Lysis Buffer (5% SDS, 50 mM Tris pH 6.5-7.0, 25 mM TCEP, 20 mM Iodoacetamide (IAC)) at a volume of 130 μL per 20 mg of tissue powder. IAC was freshly prepared from powder immediately prior to each experiment, and TCEP and IAC were added to the lysis buffer immediately before use. Immediate alkylation with IAC is essential for preserving the native cysteine acetylome. Without this blocking step, extensive S-to-S acetyl transfer occurs during sample preparation, resulting in highly variable and irreproducible acetylation patterns. All identified sites should be considered susceptible to transfer if free cysteines are available as nucleophiles. After buffer addition, samples were vortexed to ensure complete lysis and then incubated in the dark at room temperature for 20 min to allow for the alkylation of exposed cysteine residues. Following incubation, samples were centrifuged at 20,000 g for 15-20 min at 16 °C to pellet the genomic DNA. In cases where the DNA did not pellet effectively, samples were sonicated to shear the DNA before repeating the centrifugation step. The supernatant containing the proteins of interest was collected, and the pellet was discarded.

### Sample Preparation using S-Trap Micro Columns

For each sample, 25 μL of the supernatant was processed using S-Trap micro columns. 12% phosphoric acid was added at a ratio of 1:10 to the 25 μL sample (2.5 μL of 12% phosphoric acid per sample), followed by the addition of 165 μL of S-Trap buffer (90% methanol with 100 mM TEAB, pH ~7). The entire sample was loaded onto an S-Trap micro column placed in an Eppendorf tube and centrifuged at <4000 g for <3 minutes. Columns were washed 4 times with 150 μL of S-Trap buffer (90% methanol, 100 mM TEAB, pH ~7) and centrifuged at <4000 g for <3 minutes at 16 °C, discarding the flow-through after each spin. An additional spin was performed after the final wash to ensure complete removal of residual S-Trap buffer.

### Trypsin Digestion

Each S-Trap micro column was transferred to a fresh Eppendorf tube, and 20 μL of Trypsin solution containing a 1:10 (w/w) ratio of protease to protein was added directly onto each micro column. Pre-prepared Trypsin samples containing 20 μg in 40 μL total volume were used, with 20 μL (10 μg) of trypsin solution added to each column. Samples were incubated at 37 °C for 2.5 hours.

### Peptide Elution and Preparation for Mass Spectrometry

Peptides were eluted from the S-Trap micro columns in three steps: first with 40 μL of 50 mM TEAB pH 8 (added directly to the trypsin in the S-Trap), then with 40 μL of 0.2% formic acid, and finally with 40 μL of 50:50 AcN:H_2_O with 0.1% formic acid. The eluate was frozen in liquid nitrogen and dried overnight by speed vacuum. Dried samples were resuspended in 100-200 μL of 0.1% formic acid, vortexed to aid in resuspension, and incubated at room temperature to allow for complete resuspension. Samples were then centrifuged at 20,000 g for 10 minutes at 4 °C, and the supernatant was collected.

### Peptide Quantification and Mass Spectrometry Analysis

The peptide concentration of the supernatant was quantified using the Pierce Colorimetric Peptide Assay (ThermoFisher 23275). The supernatant was then transferred to mass spectrometry vials for loading and characterization by Q-Exactive Mass Spectrometry. All samples were stored at -80 °C until analysis.

### Quantitative LC/MS/MS Analysis

Label-free quantitative nanoLC-MS/MS of 1 μg of digested peptide from all experimental samples and QC pools (made from combining samples) was performed using data-dependent acquisition (DDA) on a Q Exactive Plus Orbitrap mass spectrometer (ThermoFisher Scientific) coupled to an EASY-nLC UPLC system (Thermo). For each sample, 1 μg (~3 μL) of peptide was injected and tapped on an Acclaim PepMap (Thermo) trapping (3 um, 75 µm × 20 mm) and separated on an analytical (2 um 100 C18, 75 µm × 500 mm column) column over a 105 min gradient (5 to 40% solvent B (90% ACN/0.1% FA)) at a flow rate of 300 nL/min and a column temperature of 55 °C. MS1 spectra (precursor ions) were collected at a resolution (r) of 70,000, a target AGC target value of 3e6 ions, and a maximum injection time (IT) of 100 ms. Using a cycle time of 2 s, precursor ions were selected using a 1.2 m/z isolation window and normalized collision energy of 27, with dynamic exclusion (DE) enabled for 30 s. MS2 spectra (product ions) were collected at r = 17,500, AGC=1e5, and max IT = 100 ms. All Thermo raw files were uploaded to Proteome Xchange.

### Proteomic Data Processing and Peptide Identification

Raw data for all experimental samples and QC pools were processed in Proteome Discoverer 2.5 or 3.0 (ThermoFisher). The Minora Feature Detector node was used to align precursor ion features across all runs based on mass and retention time. Relative peptide abundance was calculated using precursor intensity, considering MS1 extracted ion chromatograms (XIC) of the aligned features. Data were searched with both Sequest HT and MS Amanda 3.0 against a UniProt mouse complete proteome database (2022) and corresponding “decoy” reversed protein sequences. Considered variable modifications included oxidation (15.995 Da on M), carbamidomethyl (57.021 Da on C), and acetyl (42.011 on C, K), with up to 2 missed cleavages (full trypsin specificity). FDR for peptide spectral matches (PSMs) for each search algorithm was estimated using Percolator, after which IMP-ptmRS was used to localize modifications to specific residues. Peptide Validator was used to collapse PSMs to unique peptides at 1% FDR. Quantitation was normalized for total peptide signal within each sample, providing Normalized Abundance values to adjust for subtle differences in sample loading and LC-MS performance. Peptides were grouped to proteins (strict parsimony), which were filtered to 1% FDR with Protein FDR Validator.

### Proteomic Peptide-to-Protein Normalization

Prior to differential abundance analysis, peptide abundances were normalized to their corresponding protein abundances to account for changes in total protein expression. For each peptide-protein pair within each sample, the normalized peptide value was calculated as the ratio of peptide abundance to protein abundance. Peptides mapping to multiple proteins were treated as separate entries for each protein assignment. Peptides with zero variance across all samples (i.e., peptide and protein abundances were identical, resulting in normalized values of 1.0 across all samples) were identified and analyzed separately from peptides showing variance after normalization.

### Proteomic Data Transformation and Visualization

Normalized peptide-to-protein ratios were log2-transformed prior to statistical analysis. Data quality was assessed through distribution plots (density plots and boxplots). Principal Component Analysis (PCA) was performed on scaled and centered data using the FactoMineR package (v2.11) with visualization using factoextra (v1.0.7). Hierarchical clustering and heatmap visualization were performed using pheatmap (v1.0.12) with Pearson correlation as the distance metric and average linkage as the clustering method. For heatmaps, z-score transformation was applied to each analyte across all samples using either raw or log2-transformed data.

### Proteomic Data Differential Abundance Analysis

Differential abundance analysis was performed using the limma package (v3.58.1) in R (v4.3.2). The analysis was conducted in two parts:

1. **Peptides with variance after normalization**: These peptides showed different ratios of peptide-to-protein abundance across samples.
2. **Peptides without variance after normalization**: These peptides had identical peptide and protein abundances (normalized ratio = 1.0) across all samples and were analyzed using their original peptide abundances.

For both sets, a design matrix was created with three groups (Supernatant, Light, and Heavy fractions) and cell identity was included as a blocking factor to account for technical replicates from the same biological source. The duplicate correlation function was used to estimate the consensus correlation (correlation values: -0.018 for variant peptides, -0.093 for non-variant peptides), which was incorporated into the linear model fitting.

Contrasts were defined for three comparisons:

- Light vs Supernatant
- Heavy vs Supernatant
- Heavy vs Light

The empirical Bayes method was applied to moderate the standard errors. Results from both peptide sets were combined for each comparison, and p-values were adjusted for multiple testing using the Benjamini-Hochberg false discovery rate (FDR) correction across all peptides in each comparison.

### Proteomic Data Pathway Analysis

For gene set enrichment analysis (GSEA), peptide-level statistics were aggregated to the protein level by averaging the moderated t-statistics for peptides mapping to the same protein. Proteins were mapped to mouse Entrez gene IDs using the org.Mm.eg.db annotation package. GSEA was performed using the fgsea package (v1.29.1) with MSigDB hallmark gene sets (v2023.1.Mm). Over-representation analysis (ORA) was also conducted on specific peptide subsets using the fora function with MSigDB gene sets (v2024.1.Mm).

### Statistics

Hierarchical clustering and heatmap plotting were performed using the pheatmap R package (version 1.0.12) with correlation as the distance metric and the “average” as the clustering method. Principal Component Analysis (PCA) was performed on scaled and centered data using the R packages FactoMiner (version 2.11)^33^ and factoextra (version 1.0.7)^34^. Differential abundance analysis was performed using the limma R package (version 3.58.1)^35^. Gene set enrichment analysis (GSEA) was performed using the fgsea R package (version 1.29.1)^36^. All data wrangling and visualization were performed using the tidyverse collection of R packages (v2.0.0) in R (v4.3.2).

### Subcellular Fractionation

Mouse liver tissue was obtained as described previously. Tissue was fractionated by differential centrifugation at 70 g, 700 g, and 7,000 g into heavy membrane, light membrane, and supernatant (cytoplasmic) fractions. Samples were kept on ice. Centrifugation performed at 4 °C.

### 6 °C vs RT BAT

Age-matched mice were housed at either 6 °C or at RT. Mice were cold-adjusted by housing at 6 °C for four weeks. BAT was harvested and processed as previously described above for liver tissue.

### GAPDH in vitro acetylation

Purified GAPDH from rabbit erythrocytes commercially available from Sigma (G2267). GAPDH at 1 mg/mL was acetylated by incubation with 10 mM Acetyl-CoA in PBS at 4 °C.

### GAPDH activity assay

GAPDH activity was measured using the commercially available GAPDH Activity Assay Kit, (Sigma, MAK277).

## Supplementary information

Supplementary Figures

Supplemental Table 1

Supplemental Table 2

Supplemental Table 3

Supplemental Table legends

## Data Availability

Proteomic data is deposited on Proteome Exchange under PXD052367 (https://repository.jpostdb.org/preview/1013012298665740664062a), and Japan Proteome Standard Repository under JPST003104 (https://repository.jpostdb.org/entry/JPST003104.1).

## Code availability

Code used to analyze data available at https://github.com/hirscheylab/Cysteine-S-acetylation-is-a-post-translational-modification-involved-in-metabolic-regulation/tree/main. Datasets used for analysis available at 10.5281/zenodo.11580331 as Supplemental Datasets 1-48.
