# Supplementary material for: Cysteine S-acetylation is a widespread post-translational modification on metabolic proteins
Source: NPJ Metab Health Dis. 2025 Nov 7;3:43. doi: 10.1038/s44324-025-00081-2 (PMC12594830; doi:10.1038/s44324-025-00081-2)
Supplement: Supplementary file 1 — Supplementary Figures [file 44324_2025_81_MOESM1_ESM.pdf]

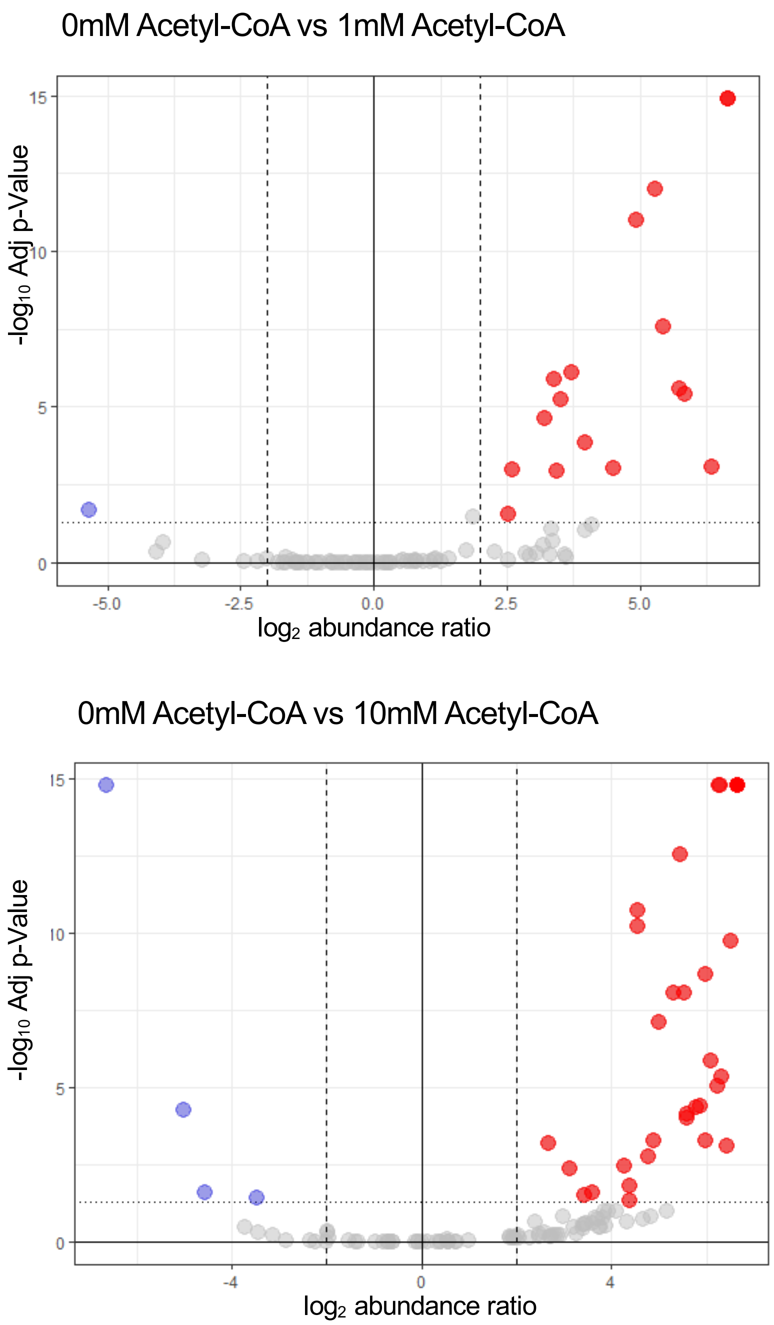

Figure S1. Acetyl-CoA treatment induces concentration-dependent increases in cysteine acetylation.

A. Volcano plots showing differential cysteine acetylation in mouse liver lysate following 1-hour incubation with acetyl-CoA at room temperature. Left panel: Comparison of 1 mM acetyl-CoA versus control (0 mM). Right panel: Comparison of 10 mM acetyl-CoA versus control (0 mM). X-axis shows log2 abundance ratio (treated/control), Y-axis shows -log10 adjusted p-value. Red dots indicate peptides with significantly increased acetylation ($p < 0.05$, fold change > 2), blue dots indicate significantly decreased acetylation, and gray dots represent peptides with no significant change. Treatment with 1 mM acetyl-CoA primarily increases acetylation at existing sites, while 10 mM acetyl-CoA recruits additional sites as evidenced by the greater number of significant hits. Dashed lines indicate cutoff thresholds for significance (vertical: 2-fold change; horizontal: $p = 0.05$).

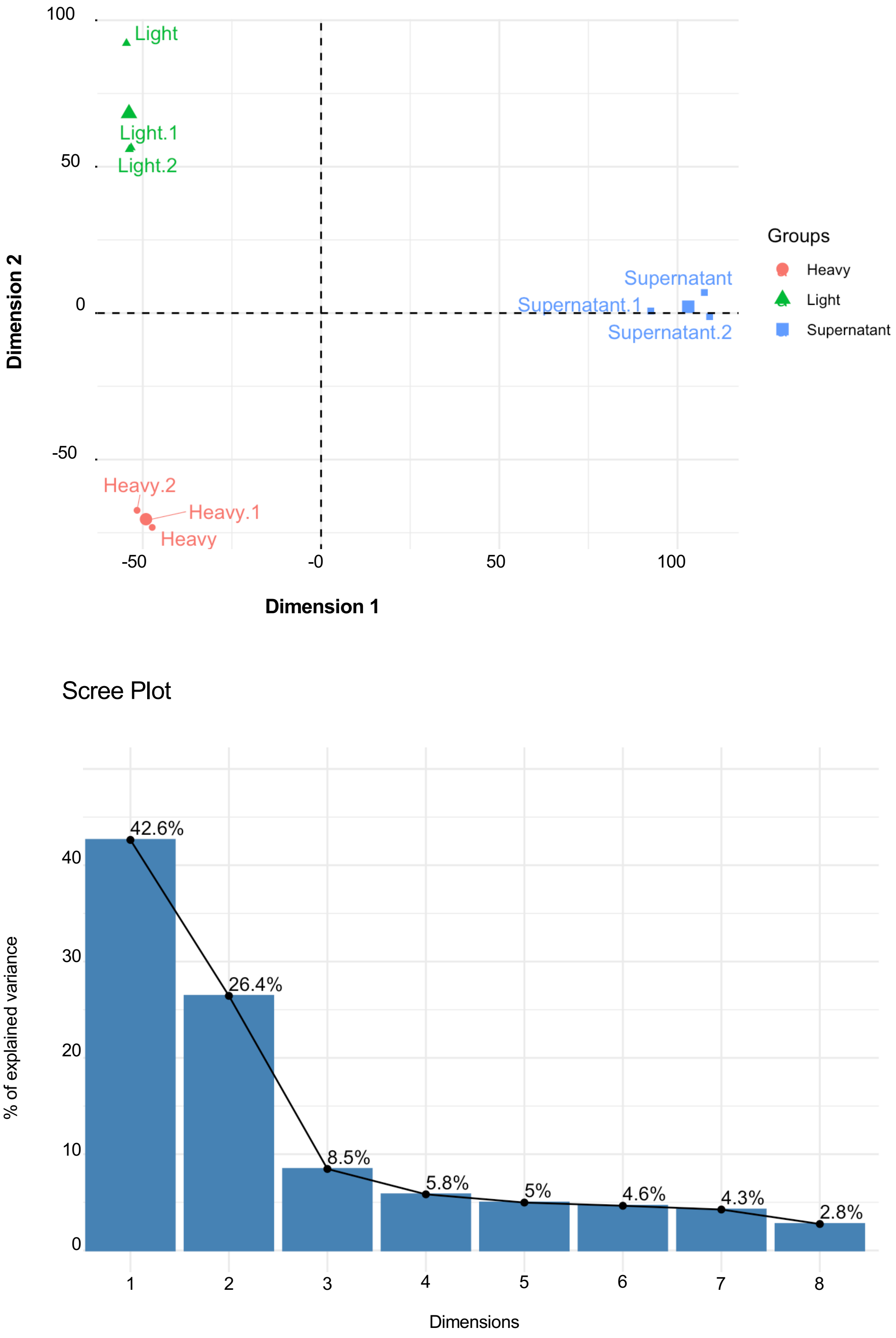

Figure S2. Analysis of the cysteine acetylome peptides following subcellular fractionation of mouse liver into heavy membrane, light membrane, and supernatant fractions.
Principal Component Analysis (PCA) of the cysteine acetylome peptides present in heavy membrane (containing mitochondria and nuclei), light membrane (containing ER and golgi), and supernatant (cytoplasmic) subcellular fractions. PC1 and PC2 account for 42.6% and 26.4% of variance, respectively. Bottom panel: Scree plot showing the relative contribution of each of the first 8 components.

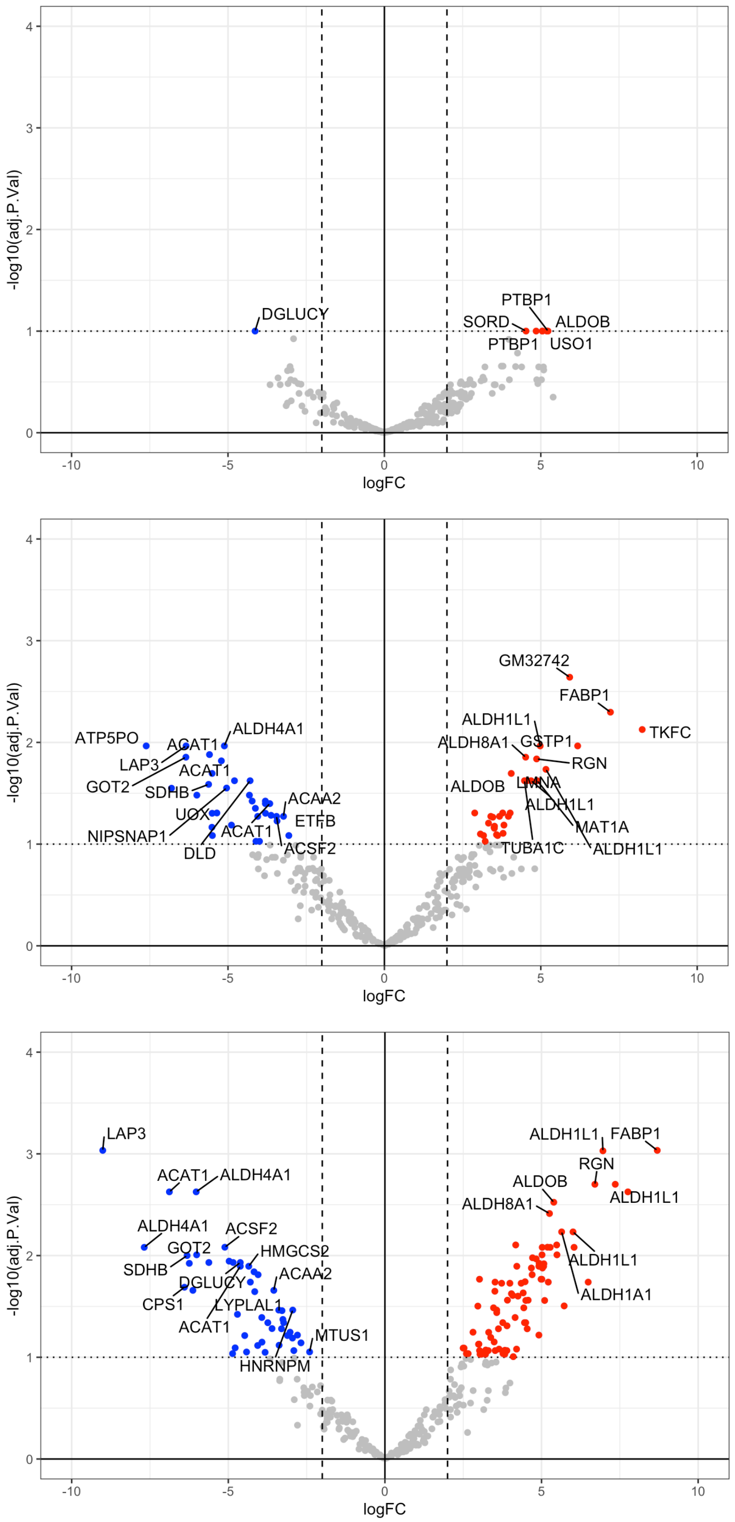

Figure S3. Proteomic validation of subcellular fractionation using organellar marker proteins. Volcano plots showing differential protein abundance between subcellular fractions from mouse liver. X-axis shows log2 fold change (logFC) and Y-axis shows -log10 adjusted p-value. Proteins enriched in each fraction are labeled and color-coded: heavy membrane markers in red, light membrane markers in blue, and supernatant (cytoplasmic) markers in blue. Dashed lines indicate significance thresholds (vertical: 2-fold change; horizontal: p = 0.05).
Top panel: Light:Heavy membrane fractions; Middle panel: Supernatant:Light membrane fractions; Bottom panel: Supernatant:Heavy membrane fractions.
