# Supplementary material for: Cysteine S-acetylation is a widespread post-translational modification on metabolic proteins
Source: NPJ Metab Health Dis. 2025 Nov 7;3:43. doi: 10.1038/s44324-025-00081-2 (PMC12594830; doi:10.1038/s44324-025-00081-2)
Supplement: Supplementary file 5 — Supplemental Table legends [file 44324_2025_81_MOESM5_ESM.docx]

**Supplemental Table 1.** Differential abundance of cysteine acetylated peptides in mouse liver following reduction with TCEP versus DTT

**Supplemental Table 2.** Cysteine acetylated peptides identified in mouse liver using immediate alkylation protocol

**Supplemental Table 3.** Dose-dependent changes in cysteine acetylation sites following treatment of mouse liver lysate with 0, 1, or 10 mM acetyl-CoA
